# Field Testing of a Virus-Particle-Based Sow Vaccine Against F4 and STb-Positive *Escherichia coli*

**DOI:** 10.3390/vaccines14060515

**Published:** 2026-06-08

**Authors:** Priscila R. Guerra, Elisabeth O. Nielsen, Ikhlaq H. Kana, Søren K. Boldsen, Vanesa García, Ana Herero-Fresno, Nicole B. Goecke, Morten A. Nielsen, Adam F. Sander, John E. Olsen

**Affiliations:** 1 Department of Veterinary and Animal Sciences, University of Copenhagen, Stigbøjlen 4, 1870 Frederiksberg, Denmark; pguerra@sund.ku.dk (P.R.G.); vanesag.menendez@usc.es (V.G.); ana.fresno@usc.es (A.H.-F.); nbgo@sund.ku.dk (N.B.G.); 2 SEGES Innovation P/S, Agro Food Park 15, 8200 Aarhus, Denmark; elin@seges.dk (E.O.N.); skbo@seges.dk (S.K.B.); 3 Centre for Medical Parasitology, Department of Immunology and Microbiology, University of Copenhagen, Blegdamsvej 3B, 2200 København, Denmark; ikhlaq@sund.ku.dk (I.H.K.); mortenn@sund.ku.dk (M.A.N.); asander@sund.ku.dk (A.F.S.); 4 Department of Microbiology and Parasitology, Faculty of Veterinary Medicine, University of Santiago de Compostella, 27002 Lugo, Spain; 5 Department of Biochemistry and Molecular Biology, Faculty of Science, University of Santiago de Compostella, 27002 Lugo, Spain; 6 Health Research Institute of Santiago de Compostela (IDIS), 15706 Santiago de Compostela, Spain

**Keywords:** enterotoxigenic *Escherichia coli*, swine, post-weaning diarrhea, virus-like particle vaccine

## Abstract

**Background/Objectives:** Post-weaning diarrhea remains a major challenge in pig production worldwide. Enterotoxigenic *Escherichia coli* (ETEC) encoding fimbriae of the F4 type and producing the heat-stable enterotoxin, STb, are one of the important causes of this disease. The aim of the current study was to evaluate whether vaccination of pregnant sows with a novel capsid virus-like particle (cVLP)-based vaccine against F4 and STb (cVLP-FaeG/cVLP-STb) could enhance performance in piglets born after such vaccinated sows. **Methods**: A field trial was conducted in a commercial sow-to-finisher pig herd. Thirty-five sows were vaccinated twice with the cVLP-FaeG/cVLP-STb vaccine prior to farrowing, while thirty-five control sows were vaccinated twice with commercial vaccines normally used in the herd. Piglets were followed until eight weeks post-weaning to assess antibody responses, diarrhea and treatment incidences, pathogen shedding, and growth performance. **Results**: Piglets born from immunized sows receiving the cVLP vaccine showed significantly higher serum antibody levels against ETEC F4 throughout the post-weaning period (*p* ≤ 0.021). The frequency of pathogen detection was similar between groups, while piglets in the cVLP group exhibited significantly lower diarrhea scores at week 6 (*p* = 0.047), showed a trend of requiring fewer treatments (*p* = 0.06) and had significantly higher final body weight (*p* = 0.048). In addition, the cVLP group showed a significantly greater average daily gain over the study period (*p* = 0.037). **Conclusion**: Sow immunization with the cVLP vaccine enhanced passive immune protection of piglets, resulting in reduced antimicrobial treatment 2 weeks post-weaning and improved growth performance.

## 1. Introduction

Post-weaning diarrhea (PWD) occurs in pigs within 14 days after weaning, affecting health, welfare and economics in affected herds [1]. In modern intensive production systems, piglets are weaned and transferred to nursery units between 21 and 28 days of age [2]. By this time, passive immunity derived from colostrum and milk has largely declined. Concurrently, the immune system of piglets is still immature, and together with the abrupt transition from a milk-based diet to solid feed, this increases the risk of infectious diarrheal disease [3,4].

The epitheliochorial placenta of the sow prevents prenatal transfer of maternal immunoglobulins during gestation [5]. Fetuses can produce their own antibodies during the late stage of gestation, but this production remains relatively immature due to limited or absent antigenic stimulation [6]. As a result, piglets rely mostly on colostrum and milk for the acquisition of antibodies after birth until their immune system is fully able to produce their own antibodies. Therefore, strategies such as sow vaccination that enhance maternal immunity prior to farrowing are important for strengthening immune protection during the early life of piglets [7,8].

Recent advances in vaccine technology have enabled the development of modular capsid virus-like particle vaccine platforms (cVLP) that use specialized anchoring strategies to display antigens in a virus-like structural context [9]. In comparative studies, cVLP vaccines have been shown to enhance immunogenicity compared to traditional subunit vaccines [10,11]. Enterotoxigenic *Escherichia coli* (ETEC) is a major causative agent of PWD. Colonization of the intestinal epithelium by ETEC is mediated by fimbriae adhesins, and in newly weaned piglets, *E. coli* expressing F4 fimbriae are particularly important. Therefore, F4 fimbriae are an important antigen of naturally acquired immunity as well as an important vaccine antigen [2,5]. In addition to fimbriae, enterotoxins, which are divided into heat-stable and heat-labile toxins, are considered important virulence factors of ETEC [1]. Recently, cVLP-based vaccines against the F4 major fimbriae protein, FaeG, and the heat-stable enterotoxin, STb, were developed [11]. Under experimental conditions, sows that were vaccinated with an FaeG-cVLP/STb-cVLP (cVLP) vaccine generated a robust IgG and IgA response against F4 fimbriae protein comparable with a commercial fimbriae-based vaccine, and it was shown that vaccine-induced antibodies were effectively transferred to piglets via colostrum [11]. However, the performance of this approach under field conditions remained untested. Therefore, the aim of the current study was to determine the production and health performances of piglets born from cVLP-vaccinated sows compared to piglets from sows receiving a traditional vaccination program, and to assess how these outcomes correlated with the antibody responses against the F4 antigen.

## 2. Materials and Methods

### 2.1. Experimental Design

The study was carried out under field conditions in a conventional Danish sow-to-finisher herd. Sows of Landrace x Yorkshire (L×Y) genetics were inseminated with Duroc (D) semen, resulting in piglet offspring with an (LxY)x D background. Seventy selected sows were earmarked and randomly divided into two groups, which were housed under identical conditions, except they were housed in separate rooms according to their assigned treatment groups. For logistical reasons, the vaccination trial was carried out with blocks of ten sows, five of each group. Both groups consisted of a mixture of first-lay (n = 18) and second-lay (n = 17) sows. Group 1 sows (n = 35) were vaccinated with a cVLP vaccine using a 2 mL syringe fitted with a long needle. The inhouse prepared vaccine formulation consisted of 300 µL F4 antigen (cVLP-FaeG) and 300 µL STb antigen (cVLP-STb) mixed with 1.0 mL of AddaVax™ (InvivoGen Europe, Toulouse, France) adjuvant, resulting in a total injection volume of 1.6 mL. Details on the vaccine have been published previously [11]. This vaccine will be referred to as the cVLP vaccine throughout the rest of this manuscript. This vaccine was administered together with a dose of Porcilis T-Brand (MSD Animal Health^® ^, Copenhagen, Denmark) containing *C. perfringens* ß-toxoid and ε-toxoid, and *C. tetani* toxoid for protection against *Clostridium perfringens*. In Group 2, sows (n = 35) received two commercial vaccines, Suisen Coli/C^®^(HIPRA, Vejen, Denmark) containing F4ab, F4ac, F5, and F6 fimbriae antigens and LT-entero-toxoid of *E. coli* and *C. perfringens* type C and *C. novyi* type B toxoids and Suiseng Diff/A^®^ (HIPRA; Vejen, Denmark) containing *Clostridioides difficile* toxoid A and B and *C. perfringens* type A toxoid, which were routinely used in the herd for active immunization against *E. coli* F4 and *C. perfringens*.

**Figure 1:**
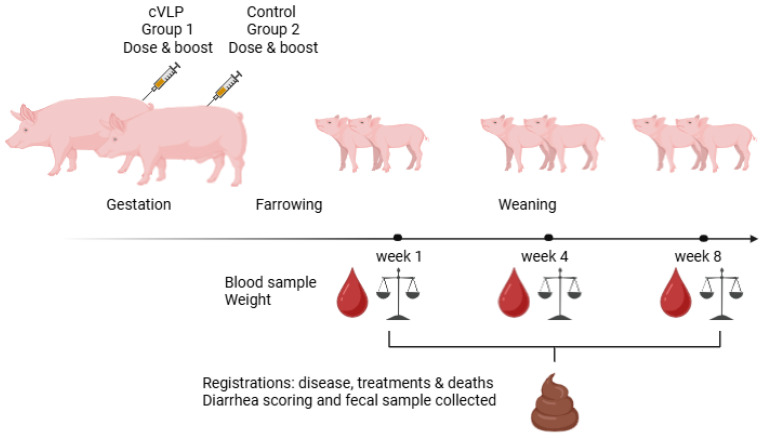
Overview of the experimental setup. The figure illustrates the study design and timeline. Seventy sows were allocated to two treatment groups. Group 1 received a novel capsid virus-like particle (cVLP) vaccine containing F4 antigen (cVLP-FaeG.Tag) and STb antigen (cVLP-STb) mixed with 0.5 mL AddaVax™ adjuvant and administered with Porcilis T-Brand^®^ (MSD Animal Health^®^). Group 2 received the commercial vaccines Suisen Coli/C^®^ and Suiseng Diff/A^®^ (HIPRA) for immunization against *E. coli* F4+ and *C. perfringens*. Sows were vaccinated six and two weeks before expected farrowing, and blood samples were collected before vaccination and two weeks after each immunization. At birth, five piglets per litter were selected and followed. Body weight and blood samples were collected at one to three days of age, at weaning (~4 weeks), and four weeks post-weaning.

Sows in both groups were vaccinated six and two weeks prior to expected farrowing. This time corresponds to the time points used in our previous experimental testing of the cVLP vaccine [11] and to the vaccination scheme normally used in the herd, where the trial was carried out. Serum samples were collected from the sows prior to vaccination and two weeks after each immunization. At birth, five piglets from each litter (average litter size: 14–15 piglets) were earmarked. These piglets represented the five individuals with body weights closest to the median weight of the litter. Piglets remained with their mothers until weaning at four weeks of age, and each sow’s offspring were subsequently followed until the age of eight weeks (four weeks post-weaning) (Figure 1). The pigs were weighed and blood samples were collected from the 5 selected piglets in each litter at one to three days of age, at weaning (approximately 4-week-old) and four weeks after weaning. Six piglets in both groups died for unknown reasons during the first and second samplings, resulting in six fewer measurement points in the following two samplings. Blood samples were collected from the jugular vein in BD Vacutainer^®^ blood collection tubes. In both groups, the number of dead pigs, the number of pigs showing signs of diarrhea and the pigs moved to sick pens were noted. Pigs showing signs of diarrhea were individually treated with an antimicrobial as per the treatment protocol in use in the herd. The protocol consists of a combination of Sultrivet 200 mg/mL (Biovet^®^, Frederiksberg, Denmark) + 40 mg/mL trimethoprim (Bela-pharm GmbH&Co.^®^, Vechta, Germany).

### 2.2. Fecal Consistency, Outbreak of Diarrhea, and Sampling for Microbiology

Pigs were inspected once a week by a technician associated with the research team. At this visit, all piglets were individually assessed for fecal consistency using a standardized scoring system. A cotton swab was gently inserted into the rectum of the piglet and fecal material adhering to the swab was scored using a four-point scoring system. A score of 1 indicated firm feces, a score of 2 indicated soft but non-liquid feces, a score of 3 indicated liquid feces, and a score of 4 indicated watery feces. Animals scoring 3 and 4 were classified as diarrheic. Daily, farm personnel inspected all pens for signs of diarrhea. In cases where more than five pigs in a pen showed diarrhea, a composite pen fecal sample and fecal-swabs-sock and rectal swabs from up to five piglets showing signs of diarrhea were taken per pen. Samples were transported under refrigerated conditions to the laboratory on the same day.

### 2.3. Vaccine-Induced Antibody Responses

Assessment of the F4 neutralizing antibody was performed using an enzyme-linked immunosorbent assay (ELISA) as previously described [11]. Briefly, microtiter plates (Nunc MaxiSorp, Invitrogen, Waltham, MA, USA) were coated with F4ac fimbriae (University of Ghent, Belgium) and recombinant FaeG. To evaluate seropositivity, all pig serum samples were run in parallel with a non-immune control serum sample (31890, Invitrogen). After washing, plates were probed with HRP-conjugated anti-pig IgG (ab6915, Abcam, Cambridge, UK) or unconjugated rabbit anti-swine IgM (SAB3700447, Sigma-Aldrich), followed by anti-rabbit IgG HRP (P0448, Agilent, Santa Clara, CA, USA). The absorbance was measured at 450 nm using a HiPo MPP-96 microplate reader (BioSan, Riga, Latvia).

### 2.4. qPCR and Culturing of Fecal Swabs

Based on composite pen (fecal sock) samples, ETEC F4+, F18+, rotavirus A and C, *Brachyspira pilosicoli*, and *Lawsonia intracellularis* were quantitatively detected using a high-throughput real-time PCR system (qPCR), including relevant negative and positive controls, as previously described [12]. Rectal swabs from individual pigs suffering from diarrhea were cultured on blood agar plates (Oxoid blood agar base III supplemented with 5% cattle blood) and incubated overnight at 37 °C. Shedding of hemolytic *E. coli* was assessed, and the presence of fimbriae and toxin types of ETEC was determined by multiplex PCR analysis, as previously described [13].

### 2.5. Statistical Analysis

The data were analyzed as a cohort study to assess differences between the two experimental groups. A significant level of 5% was applied for all statistical tests. Statistical analyses were performed using generalized mixed-effects models in R: 4.1.2 using lmer and glmer functions (binomial distribution) from lmerTest 1.1-32 and the lme4 package 3.1-3. In all models, sows nested in farrowing batches were included as a random effect to account for clustering within litter. For analyses of measurements obtained after weaning, pen was additionally included as a random effect. Apart from body weight in the first week, which was included as a linear explanatory variable, all other outcomes were analyzed without adjustment for baseline weight.

### 2.6. Ethical Statement

The field trial was carried out as a clinical trial with permission from the Danish Medicines Agency (case 2023122017). The testing was carried out in adherence to Danish guidelines encompassing guidelines for animal welfare established by FELASA (Federation of European Laboratory Animal Science Associations).

## 3. Results

### 3.1. Induction of Immune Response in Sows and Transfer of Immunity to Offspring

Thirty-five sows were vaccinated with the test and control vaccines. One sow in the test (cVLP) group died during farrowing. Firstly, the induced immune response in sows vaccinated twice before farrowing with the cVLP vaccine was compared to the response in sows vaccinated twice with the traditional sow vaccine regime used in the herd. This was done by measuring the specific antibodies to the F4 component of the vaccine. Both vaccine strategies induced a strong response in sows toward F4 fimbriae (Supplementary Table S1 with graphical overview in Supplementary Figure S1). The average OD_450_ value of sows vaccinated with the cVLP vaccine after immunization was 1.27 (SEM 0.09), which was considerably and significantly (*p* < 0.001) above the value of 0.69 (SEM 0.08) measured in the traditionally vaccinated group.

Next, the extent to which antibodies were passively transferred to piglets through colostrum and milk was determined. Piglets in both groups had the highest antibody levels at one week of age and the level declined towards the end of the study after eight weeks. During the full test period (week 1, week 4 and week 8), piglets born from cVLP-vaccinated sows showed a significantly higher antibody response to *E. coli* F4 than offspring from the traditionally vaccinated sows. At both week 1 and week 4, the minimum OD_450_ value observed in any pig in the cVLP-vaccinated group was above the maximum value observed in any pig in the traditionally vaccinated group, while a slight overlap was observed between the two groups at the end of the experiment (week 8) (Table 1 and Table S1).

### 3.2. Health and Growth Performance of Piglets

To evaluate the health and growth performance of piglets, diarrhea scores post-weaning, number of treatments in the nursery unit, and body weight gain of piglets were monitored and compared between groups. A similar proportion of piglets in the cVLP and the control group had diarrheal scores of 3 or 4 during the first and the last week post-weaning. In the second week post-weaning (week 6), however, a significantly lower proportion of the pigs in the cVLP groups had these high fecal consistency scores (*p* = 0.047) (Table 2). Additionally, piglets in the cVLP group required fewer pen treatments (n = 0.30 per day) compared with control piglets (n = 0.35 per day), although this difference was not statistically significant (*p* = 0.056) (Table 2 with full data in Supplementary Table S2).

Body weights were similar across all experimental groups at week 1 and week 4, with no statistically significant differences observed at these early time points (*p* = 0.551 and *p* = 0.570, respectively), which indicated a similar baseline weight and similar growth performance until weaning. In contrast, by week 8, piglets from the cVLP group were significantly heavier (*p* = 0.048), weighing almost 1 kg more than pigs in the control group (Table 2 with detailed data in Supplementary Table S2). Thus, from weeks 4 to 8, piglets born from cVLP-vaccinated sows had a significantly higher ADG of 214.73 g compared to 178.59 g for piglets in the control group (*p* = 0.003). Similarly, the ADG calculated over the entire period from week 1 to 8 was also significantly higher in the cVLP group than in the control group (*p* = 0.037) (Table 2). These results indicate that growth-performance advantages became more pronounced after the initial post-weaning adaptation phase and were sustained over time.

### 3.3. Detection of Enteric Pathogens in Composite (Fecal-Sock Samples) and Rectal Samples of Pigs Showing Signs of Diarrhea

The cVLP vaccine contained antigens against the two important ETEC virulence factors, F4 and STb. Microbiological follow-up was performed using both culture-based detection of hemolytic *E. coli* and PCR-based characterization of *E. coli* based on individual rectal swab samples, as well as qPCR and RT-qPCR analysis targeting major enteric bacterial pathogens and rotavirus A and C in 180 composite (fecal-sock) samples. Eighty-six of the sock samples originated from the cVLP group and 94 from the control group. RT-qPCR analysis on the fecal-sock samples demonstrated *E. coli* F4 gene-target in 30/86 (34.9%) of the samples in the cVLP group and 23/94 (24.5%) in the control group (*p* = 0.142 by Fisher’s exact test). The median C_t_ values of positive samples were 17.3 and 21.8, respectively (*p* = 0.078 by Mann–Whitney rank-test). The STb-toxin gene was detected in 49/86 (56.9%) of the samples originating from the cVLP group and in 59/94 (62.8) samples from the control group (*p* = 0.450 by Fisher’s exact test) with the corresponding median C_t_ values of 20.5 and 20.3 (*p* = 0.713 by Mann–Whitney rank-test (data in Supplementary Table S3). In addition to F4 and STb gene-positive ETEC, samples positive for ETEC F18+, *B. pilosicoli* and rotavirus A were identified in samples from both groups, indicating concurrent exposure to multiple enteric pathogens during the post-weaning period.

Two hundred and forty-seven diarrheal fecal samples were analyzed. Of these samples, 111 originated from pigs with diarrhea in the cVLP group and 136 samples from pigs in the control group. Hemolytic *E. coli* was detected in 38.7% (n = 43) of the samples in the cVLP group and 29.4% (n = 40) in the control group (*p*= 0.91) (Table 3). Among isolates recovered from these samples, 14 isolates were found to be F4 positive in the cVLP group and seven in the control group (*p* = 0.65). Similarly, nine of the isolates in the cVLP group were STb positive by PCR while 17 were STB positive in the control group (*p* = 0.50).

## 4. Discussion

The postweaning period is a critical window of vulnerability in the life of piglets, underscoring the need for strategies that enhance early immune protection and intestinal health. ETEC remains the primary etiological cause of PWD in piglets and constitutes a major driver for antimicrobial usage in swine production [14]. Therefore, identifying preventive strategies aimed at strengthening immune protection during this critical post-weaning transition is of paramount importance to improve pig health and enhance production efficiency. F4 fimbriae and the STb enterotoxin represent major virulence factors of ETEC [13], with F4 serving as a key antigen mediating naturally acquired immunity [5].

VLP-based vaccines have emerged as promising platforms in human medicine due to their safety, good antigenicity and immunogenicity [10]. More recently, their application has expanded into veterinary medicine, in which they have shown good performance as preventive measures against a range of bacterial and viral pathogens [15,16]. In a previous study, the cVLP construct was evaluated in the current field trial booster immunization of sows, leading to efficient maternal antibody transfer to piglets via colostrum [11]. These findings prompted us to investigate whether maternal immunization with the cVLP vaccine during gestation would enhance post-weaning performance and increase anti-*E. coli* F4 IgG levels in piglets, compared with offspring of sows vaccinated with a control *E. coli* vaccine under field conditions.

Immune globulin levels in neonatal piglets predominantly represent maternal antibodies acquired via colostrum and milk, subsequently absorbed and contributing to local intestinal immunity. Colostrum intake within the first 24 h after birth is crucial for early-life survival, as piglets rely heavily on maternally derived immune defenses to support optimal growth, development and protection against early-life infections [6,17,18]. Antibody concentrations in sow colostrum serve as a key indicator of the systemic immunity transferred to the piglets, and under experimental conditions, the cVLP vaccine tested in this study induced higher levels of both IgG and IgA in sow milk compared with a traditional vaccine [11]. Due to logistical constraints, colostrum samples were not available for analysis during this field study, and sow IgG levels were therefore used as an indicator of the immune status of the sows. As previously observed under experimental conditions [11], sows vaccinated with the cVLP vaccine exhibited higher serum IgG levels at parturition compared with sows receiving a traditional/conventional vaccine. These findings indicate that sows vaccinated with the cVLP vaccine were likely to confer higher levels of protective antibodies to their offspring than sows in the control group. In line with this, piglets from the cVLP group demonstrated significantly elevated serum antibody levels against the F4 antigen throughout the trial period relative to control piglets. As expected, antibody levels in piglets in both groups declined progressively following weaning, reflecting the natural decay of passively acquired maternal immunity [17]. Our focus on F4-directed antibodies rather than the other vaccine antigen, STb, was guided by previous results, which showed that, in general, antibody levels against STb were lower than those detected against F4 [11].

Antimicrobial resistance is a global health concern [19]. At the population level, the prevalence of antimicrobial resistance is directly correlated with the frequency of antimicrobial treatments [20]. In Danish pig herds, the highest incidences of antimicrobial treatment occur during the first two weeks post-weaning [21]. Despite not being significant (*p* = 0.056), it was encouraging to observe that piglets in the cVLP group showed a trend to have lower overall treatment incidences and a lower incidence of diarrhea in the second week after weaning compared with the control group.

High-quality colostrum intake has been linked not only to improved immunity but also to enhanced daily weight gain and overall growth performance, highlighting the broad benefits of effective lactogenic support during the neonatal period [22,23]. For unknown reasons, vaccination with a cVLP vaccine targeting *Lawsonia* *intracellular* resulted in a reduced weight gain in vaccinated mice, although the underlying mechanisms remain unknown [16]. From a production perspective, a similar outcome in pigs would render such a vaccination strategy ineffective, except in cases in which the vaccine confers protection against critical diseases such as level A contagious diseases. Interestingly, in our study the higher antibody levels in piglets from cVLP-vaccinated sows were associated not only with improved overall health, as reflected by the reduced treatment incidences, but also with increased average daily weight gain, resulting in piglets weighing almost 1 kg more on average than those in the control group at week 8. This suggests a potential economic benefit of the vaccine. These findings are consistent with previous studies demonstrating that effective passive immune transfer enhances piglet resilience to enteric pathogens and supports robustness during post-weaning periods [5,24,25]. Notably, performance differences became more pronounced during the later post-weaning period, a stage when maternal antibody protection gradually declines and piglets increasingly rely on the development of their own immune system. This underscores that improved health status and reduced disease burden during early life are likely to contribute to enhanced resilience and productive performance during the post-weaning period. PWD exhibits herd-specific characteristics regarding etiology and underlying management factors [26]. Therefore, further testing across different herds will be necessary to determine whether the observed benefits represent a general feature of the vaccine and vaccination strategy, rather than a herd-specific effect, and whether the differences will remain through the finishing period.

A clear weakness of the current study was that antibody response to the STb component of the vaccine was not measured. This was due to technical constraints. The prevalence of hemolytic *E. coli*, which is commonly associated with ETEC, in pigs suffering from PWD [14] did not differ between the test and control groups. Similarly, both analyses of rectal swabs and fecal-sock samples indicated that shedding of F4+ ETEC and STb-positive ETEC was not different between groups. This suggests that the maternal vaccination strategy, which reduced treatment incidences and improved growth performance, may primarily function by reducing the clinical severity of disease and enhancing piglet resilience, rather than by preventing bacterial colonization or environmental transmission. Due to the lack of information on STb responses, we could not determine which antigen was more likely to be responsible for this observation. Also, we cannot rule out that the effects of the vaccination may be mediated through mechanisms independent of specific protection against F4^+^/STb-positive ETEC, as the current field study design does not permit differentiation between these mechanisms.

A range of different ETEC strains and other intestinal pathogens such as *B. pilosicoli* and rotavirus A were detected in fecal-sock and rectal swabs from diarrheic pigs. The vaccine formulation used in the current study consisted of a combination of two cVLPs, each displaying one of the two vaccine antigens, FaeG or STb. A notable feature of the cVLP platform is its flexibility for constructing multi-antigen vaccines, either by displaying multiple antigens on the same capsid or as applied in the current study, by mixing identical capsids displaying different antigens. Regarding PWD, it would be valuable to develop and evaluate vaccines that, in addition to the F4 and STb antigens targeted in this study, incorporate cVLPs displaying immunogenic protein from rotavirus A, *B. pilosicoli,* and *L. intracellularis*, as well as antigens corresponding to F18 adhesin and the STa and LT toxins.

## 5. Conclusions

Maternal immunization of sows with a cVLP vaccine against ETEC F4 and STb enhances the passive transfer of immunity to piglets, resulting in tangible improvements in health and performance post-weaning. Compared with control piglets, those born to cVLP-vaccinated sows demonstrated elevated antibody titers, tended to require fewer antimicrobial treatments, and exhibited superior growth during the post-weaning period.

## Figures and Tables

**Table 1:** Serum antibodies (OD_450_ nm) against *E. coli* F4 fimbriae protein, FaeG, in piglets born after cVLP-vaccinated sows and piglets born after sows vaccinated with traditional vaccines ^1^.

	Commercially Vaccinated Group			cVLP Vaccinated Group			*p*-Value ^2^
	Average	Minimum	Maximum	Average	Minimum	Maximum	
Week 1	0.810	0.671	0.977	1.503	1.236	1.828	0.000
Week 4	0.433	0.334	0.562	0.792	0.605	1.036	0.001
Week 8	0.247	0.172	0.357	0.453	0.317	0.646	0.021

^1^ See Section 2 (Materials and Methods) for a description of vaccination strategies. ^2^ *p*-value for the null hypothesis that the average antibody levels were not statistically different between the two groups.

**Table 2:** Proportion of pigs with diarrhea and growth performance of pigs per week during the post-weaning period in piglets born after cVLP-vaccinated sows and piglets born after sows vaccinated with traditional vaccines ^1^.

	Control Group			cVLP Group			*p*-Value ^2^
	Average	Minimum	Maximum	Average	Minimum	Maximum	
Diarrheal score week 5 ^3^	35.75	18.40	57.85	35.73	18.24	58.09	0.998
Diarrheal score week 6	24.12	9.03	50.46	11.01	3.32	30.80	0.047
Diarrheal score week 7	30.51	17.91	46.90	31.75	18.64	48.56	0.832
Diarrheal score week 8	21.10	12.09	34.21	19.24	10.73	32.09	0.747
Treatments week 5–8 ^4^	0.35			0.30			0.056
Weight Week 1 (kg)	1.86	1.74	1.99	1.91	1.79	2.04	0.551
Weight Week 4 (kg)	7.00	6.60	7.41	6.86	6.44	7.28	0.570
Weight Week 8 (kg)	11.96	10.99	12.92	12.94	11.98	13.91	0.048
Average daily weight gain (g) weeks 4 to 8	178.59	148.85	208.33	214.73	184.99	244.47	0.003
Average daily weight gain (g) weeks 1 to 8	208.61	189.45	227.76	229.40	210.20	248.61	0.037

^1^ See Section 2 (Materials and Methods) for a description of vaccination strategies. ^2^ *p*-value for the null hypothesis that the average per line of the two groups is not statistically different. ^3^ % of pigs with a diarrhea score above 2. ^4^ Number of antimicrobial treatments per day.

**Table 3:** Culture-based detection of hemolytic *E. coli* and ETEC virulence genes in 247 samples from piglets with diarrhea among piglets born after cVLP-vaccinated sows and piglets born after sows vaccinated with traditional vaccines ^1^.

	cVLP Group (n = 111)	Control Group (n = 136)	*p*-Value ^2^
Hemolytic *E. coli*	43 (38.7%)	40 (29.4%)	0.91
F4 positive ^3^	14 (12.6%)	7 (5.2%)	0.65
STb positive ^3^	9 (8.1%)	17 (12.5%)	0.50

^1^ See Section 2 (Materials and Methods) for a description of vaccination strategies. ^2^ *p*-value for Fisher’s exact test comparing the frequency of hemolytic *E. coli*, F4 and STb-positive samples between the cVLP and control groups. ^3^ Number of positive isolates out of the hemolytic *E. coli*.

## Data Availability

All underlying data for the current study are presented in the manuscript or as part of the Supplementary Material.

## Supplementary Materials

The following supporting information can be downloaded at: https://www.mdpi.com/article/10.3390/vaccines14060515/s1, Figure S1: Graphic overview of Elisa results (OD450 values) in sow and piglets. Table S1: Serum antibodies against F4 antigen in individual sows and piglets; Table S2: Weight and diarrhoea scores per piglet. Table S3: Overview of microbiological results.

## Abbreviations

The following abbreviations are used in this manuscript:

ETEC	Enterotoxigenic *Escherichia coli*
cVLP	Virus-like protein-based vaccine
F4	Fimbriae type 4
F18	Fimbriae type 18
STa/STb	Heat-stable Enterotoxin
qRT-PCR	Quantitative Reverse Transcription Polymerase Chain Reaction
IgA/IgG	Immunoglobulin A and G
HRP	Horseradish Peroxidase
PWD	Post-Weaning Diarrhea
ELISA	Enzyme-linked immunosorbent assay
