# Supplementary material for: Field Testing of a Virus-Particle-Based Sow Vaccine Against F4 and STb-Positive Escherichia coli
Source: Vaccines (Basel). 2026 Jun 8;14(6):515. doi: 10.3390/vaccines14060515 (PMC13307905; doi:10.3390/vaccines14060515)
Supplement: Supplementary file 1 [file vaccines-14-00515-s001.zip › Supplemental Figure S1.pdf]

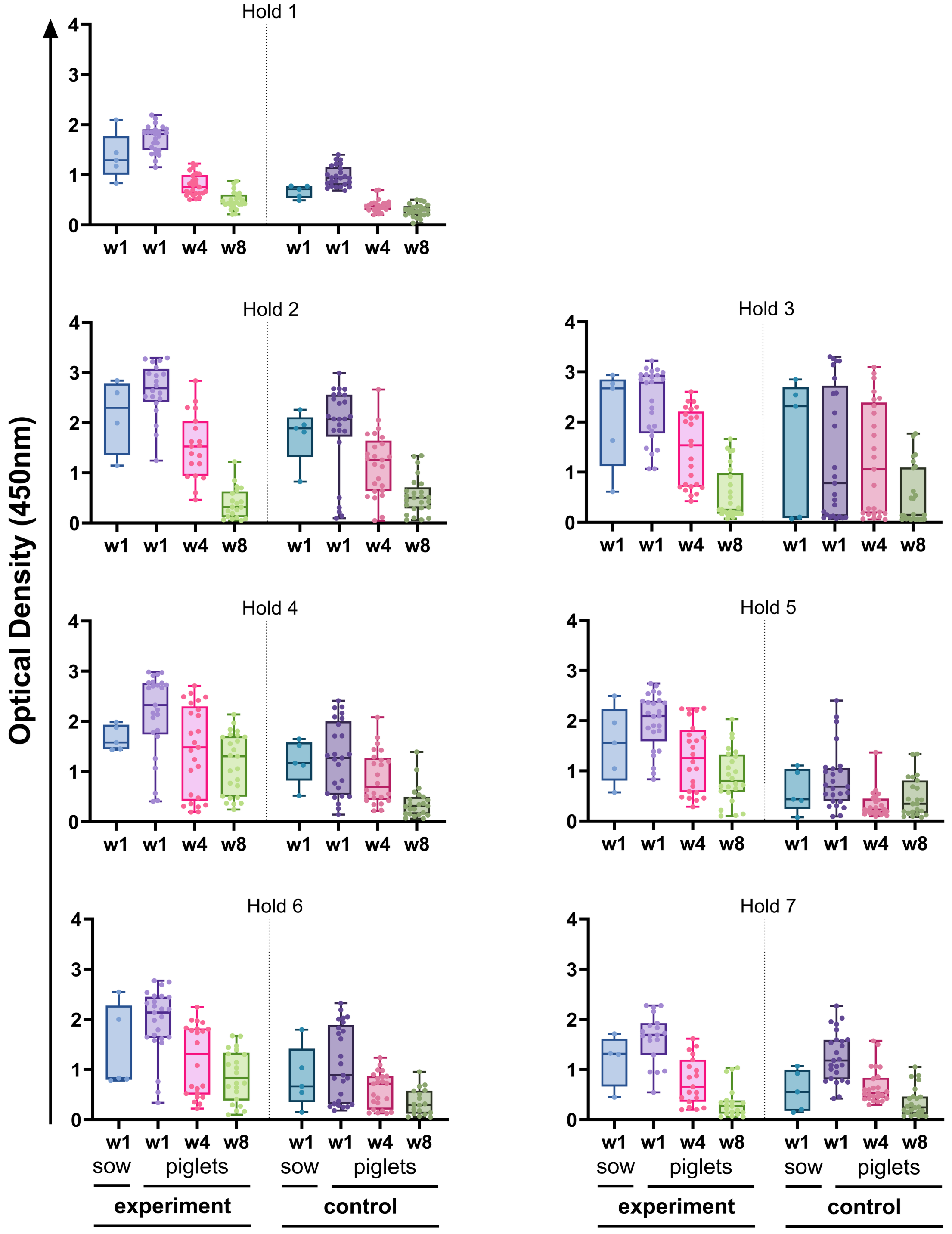


Supplemental Figure S1. Graphic overview of Elisa results (OD450 values) in sow and piglets. Hold 1-7 refers to the seven baches in which the vaccine trial was carried out.
